# Development and validation of a new comprehensive measurement tool for health insurance literacy

**DOI:** 10.1186/s12913-025-12881-9

**Published:** 2025-12-08

**Authors:** Reut Ron, Moriah E. Ellen, Paula Feder-Bubis

**Affiliations:** 1 Department of Health Policy and Management, Faculty of Health Sciences, Ben-Gurion University of the Negev, P.O.B. 653, Beer-Sheva, 8410501 Israel; 2 Assuta Health Research Institute, HaBarzel 20, Tel Aviv, Israel; 3 Department of Health Policy Management and Evaluation, University of Toronto Dalla Lana School of Public Health, Toronto, ON Canada

**Keywords:** Health insurance literacy (HIL), Measurement tool development, Questionnaire validation, Health insurance literacy measure (HILM), Cultural adaptation, Healthcare systems, Public health, Health policy, Survey

## Abstract

**Background:**

Health insurance literacy (HIL) reflects individuals’ ability to understand, select, and effectively use health insurance, impacting healthcare access and utilization. Existing measurement tools often lack comprehensiveness or contextual relevance. This study aimed to develop a comprehensive questionnaire for measuring HIL, encompassing all recognized dimensions and expanding upon the Health Insurance Literacy Measure (HILM), while also adapting it to the specific cultural and national context, using Israel as a case study and proof of concept.

**Methods:**

A multi-phase methodology was employed to develop a comprehensive HIL questionnaire, including an extensive literature review, expert consultations, and iterative pilot testing to ensure cultural and contextual relevance. Exploratory factor analysis (EFA) and reliability testing were conducted using data from a representative sample of 1,012 adults to validate its psychometric properties.

**Results:**

A 75-item questionnaire was designed, covering four domains: confidence and behavior in choosing and using health insurance (HILM), self-report confidence in understanding of key insurance concepts, objective knowledge assessment, and self-assessment of HIL. The questionnaire employs a combination of Likert-type scales and binary scoring for objective knowledge items. EFA confirmed a robust multidimensional structure. The final model accounted for 61% of the variance in the confidence and behavior domains and 57% in the concept domain, while the objective knowledge domain showed less definitive factor loadings. Internal consistency was high across all domains (Cronbach’s alpha = 0.80–0.95), and concurrent and convergent validity analyses demonstrated moderate to strong correlations with external measures of understanding and self-assessed knowledge, supporting its psychometric robustness.

**Conclusions:**

This validated questionnaire presents a robust, culturally adapted measure of HIL, integrating both objective knowledge and subjective confidence, offering insights into the multidimensional nature of HIL. It provides critical insights for policymakers and educators aiming to enhance public understanding and effective use of health insurance, setting the stage for targeted interventions and broader international applications.

**Trial registration:**

Not applicable.

**Supplementary Information:**

The online version contains supplementary material available at 10.1186/s12913-025-12881-9.

## Background

Health insurance literacy (HIL) refers to individuals’ ability to understand, obtain, and effectively utilize health insurance, enabling informed decision-making and efficient navigation of complex insurance systems [7, 9, 13, 18]. A growing body of research highlights the complexity of HIL, which encompasses not only self-reported confidence and behaviors but also objective knowledge, comprehension of key insurance concepts, and the ability to effectively apply this knowledge in real-world decision-making [8].

Several measurement tools have been developed to assess HIL, most notably the Health Insurance Literacy Measure (HILM), which evaluates individuals’ confidence and behaviors in selecting and using health insurance [16]. Recently, the HILM questionnaire was adapted for use in Switzerland [2] and the Netherlands [11], demonstrating its applicability in diverse cultural contexts and health systems.

While HILM primarily measures self-assessed confidence and expected behaviors, studies have shown that individuals frequently overestimate their own HIL, leading to poor decision-making and suboptimal healthcare utilization. Research by the American Institutes for Research found significant gaps between perceived and actual HIL, highlighting that only 20% accurately estimated costs despite high self-confidence [15]. Similarly, studies among college students and vulnerable populations have documented significant discrepancies between perceived and actual knowledge, reinforcing the need for tools that incorporate both subjective and objective measures of literacy [12]. Expanding and enhancing the measurement tool to include these additional dimensions provides a more accurate and comprehensive assessment of HIL and its impact on healthcare decision-making. Studies have shown that different metrics and questionnaires for assessing HIL may capture various concepts and yield diverse results [5].

HIL includes universal skills, such as understanding policy terms, evaluating coverage options, and calculating costs, along with local elements reflecting specific health system structures and cultural contexts [3, 14, 20]. A review of HIL assessment tools emphasizes the importance of adapting questionnaires to diverse populations and systems to ensure validity and reliability [19]. These differences underscore the importance of developing HIL measurement tools that include culturally and system-specific factors while maintaining core competencies to ensure comparability and global applicability [14].

A literature review on assessment tools for measuring HIL [19] conducted a comprehensive evaluation of the tools used to measure individuals’ ability to understand, choose, and use health insurance. The review analyzed the validity, reliability, and usability of each questionnaire, and differentiated between self-report questionnaires and knowledge test questionnaires. The review emphasizes the importance of adapting the tools to different populations and health systems to achieve an accurate and reliable assessment of the HIL level of respondents and the studied population.

The Israeli healthcare system operates under a universal and mandatory health insurance framework, established by the National Health Insurance Law of 1995, ensuring all citizens and residents receive a standard benefits package through one of four non-profit health maintenance organizations (HMOs). Beyond this basic coverage, many opt for supplementary insurance offered by the HMOs or private insurance plans, providing enhanced or additional services. However, overlapping coverage between public, supplementary, and private insurance often causes confusion and underutilization of benefits [6].

This study aims to (1) expand the HILM framework by incorporating objective dimensions of knowledge and comprehension, and (2) adapt and validate an enhanced HIL questionnaire, bridging global standards and local contexts, with Israel serving as a case study and pilot setting. The resulting tool will enable accurate assessment of HIL and facilitate improved healthcare decision-making.

## Methods

The development and validation of the new questionnaire involved three sequential stages: (1) *questionnaire* development, (2) content and structural validation, and (3) statistical validation. This section briefly outlines all three stages, while the results section specifically presents findings from statistical validation.

### Stage 1: questionnaire development

The development of the new HIL questionnaire began with an extensive literature review examining existing self-administered instruments measuring HIL across various international contexts. Building upon the established HILM questionnaire, which primarily assesses confidence and behavior in choosing and using health insurance, we expanded the questionnaire by incorporating additional critical dimensions of HIL that were not captured in the original HILM. Specifically, we added dimensions evaluating respondents’ self-reported conceptual understanding of key health insurance terms, respondents’ objective knowledge regarding insurance-related factual information, and their general self-assessment of overall HIL. The final questionnaire comprised 75 questions organized into six dimensions, as presented in Table 1. The English language version of the new developed “Israeli Health Insurance Literacy Questionnaire” is provided in a supplementary file.

The HILM questionnaire was translated and adapted into Hebrew to align with the Israeli healthcare system while preserving consistency with international versions for cross-national comparisons. A detailed review of the English-US, Dutch-Netherlands, and German-Switzerland versions informed the adaptation process, ensuring the relevance of selected questions for the Israeli context. The translation utilized a back-translation approach supported by AI tools (Google Translate, ChatGPT, and Claude.ai).

**Table 1:** Structure and dimensions of the final questionnaire

	Dimension	No of questions	Scale
1	HILM	21	4-point scale similar to the original version
2	Self-report confidence in understanding of key insurance concepts	11	(1) I have never heard of this term (2) I have heard of this term but do not understand it (3) I understand this term in context, but cannot explain it (4) I understand this term well and can explain its meaning
3	Objective knowledge assessment	23	(1) True (2) False (3) I don’t know
4	Self-assessment of HIL level	1	1–10
5	Data on purchase and utilization of health insurance	3	Changing scales
6	Demographic information	16	Changing scales

Significant modifications were made to reflect Israel’s unique healthcare model. For instance, a question about seeking help to afford insurance outside an employer was omitted due to Israel’s universal health insurance system. Instead, a new question was introduced to address the distinction between supplementary HMO’s insurance and private commercial policies. Additionally, questions such as verifying which doctor is working in the HMO, was expanded to include checking treatment coverage prior to care, reflecting a broader focus in the Israeli version compared to the Dutch and Swiss adaptations. A comprehensive comparison of all four versions is provided in the appendices.

During the questionnaire development process, careful attention was given to ensuring that both the instructions and the questions were written in plain, clear, and accessible language. This approach aimed to make the questionnaire easy to understand and to minimize the potential for misinterpretation by respondents. Additionally, answer options were designed to be straightforward and inclusive, with the inclusion of “I don’t know” as an additional response choice. This option was provided to reduce respondents’ likelihood of guessing when they were uncertain about or did not know the correct answer.

### Stage 2: content and structural validation

The complete questionnaire (all six dimensions) was subjected to an initial validation process, starting with content validation. The questionnaire was reviewed by six experts in the field of health insurance in Israel, including academics and researchers with extensive experience in HIL and health policy research. These experts have previously contributed to the development of similar instruments tailored for the Israeli population. Their feedback led to structural refinements, such as clearer dimensions divisions, minor modifications to terminology to improve cultural appropriateness, and the inclusion of additional items to address knowledge gaps regarding health coverage and policy regulations.

In parallel, a pilot study was conducted with 52 insured individuals from diverse demographic backgrounds to ensure the questionnaire’s comprehensibility and usability. Participants were asked to complete the questionnaire and provide structured and open-ended feedback regarding its length, clarity, and ease of completion. The results indicated that while 52% of respondents found the questionnaire to be reasonable in length, 40% expressed concerns about its length, and 8% found it excessively long. Additionally, 85% of participants reported that the questions were clear, while 15% identified some questions as complex or unclear. Open-ended feedback highlighted several areas for improvement, including the need to streamline certain questions, enhance mobile compatibility, and refine the wording of some response scales.

Following the content validation, structural validation was conducted. An expert in survey design was consulted to review the questionnaire’s structure. The feedback provided related to the order of questions, the clarity of wording, and the organization of response scales. The recommendations were used to refine the structure, improving the logical flow and ensuring that the questionnaire was easy to understand and complete. This process also helped minimize potential biases that could arise from confusing or poorly structured questions.

Based on both experts’ reviews and pilot study feedback, modifications were made to improve readability, optimize mobile accessibility, and clarify question phrasing. Specific suggestions, such as incorporating a more standardized Likert scale across dimensions and refining the classification of insurance types, were implemented to enhance the validity and reliability of the instrument. These refinements ensured that the questionnaire adequately captured all relevant aspects of HIL in the Israeli context while maintaining clarity and methodological rigor.

To ensure inclusivity and accessibility across Israel’s diverse population, the questionnaire was translated into Arabic and Russian (the two major language minorities use in Israel) using a meticulous back-translation process. In each language, two native speakers independently reviewed the translated questionnaire carefully examining the linguistic nuances and cultural appropriateness of the translated items. These native speakers, who were not involved in the initial translation, conducted separate reviews to validate the translation’s accuracy, clarity, and cultural relevance, ensuring that the questionnaire maintained its conceptual integrity across different language contexts.

### Stage 3: statistical validation

The questionnaire was distributed online to a representative sample of the Israeli population. Participants were recruited through a well-established, nationally representative online panel (iPanel) comprising approximately 100,000 panelists. Random sampling was conducted by sending email and text-message invitations to randomly selected panelists within predefined demographic strata, including sectors (Israeli-born Jews, immigrant Jews, Arabs), age groups, geographic regions, and socioeconomic status. Quotas were set according to population proportions to achieve representativeness of the adult Israeli population, ensuring generalizability of the findings. Each participant could choose at the beginning of the survey whether to respond in Hebrew, Russian, or Arabic.

Participants were targeted from the age of 21 and older rather than 18, as younger individuals in Israel (18–20) typically receive health insurance coverage through compulsory military service or remain dependents under parental insurance plans. Consequently, their limited direct experience with independently selecting or utilizing health insurance plans justified the decision to exclude this age group from the study.

Internal consistency was measured using Cronbach’s alpha and average inter-item correlations. In addition, exploratory factor analysis (EFA) was conducted, by preliminary tests to confirm suitability: Bartlett’s test of sphericity and the Kaiser-Meyer-Olkin (KMO) measure of sampling adequacy, which ensure the dataset is appropriate for factor analysis.

Validity was assessed through group comparisons, examining mean scores across five participant categories based on their self-reported use of supplementary and private commercial health insurance over the past year. The groups were categorized to reflect varying levels of health insurance engagement, from active use of both types to no recent use or not using at all. It was hypothesized that more active users would exhibit higher HIL scores. Analysis of variance (ANOVA) was applied to test for significant differences between groups, aligning with methodologies from the original HILM validation in the United States and the Dutch adaptation by Holst et al. [11].

Concurrent and convergent validity were assessed by calculating correlations between the different dimensions of the questionnaire. Concurrent validity was examined by comparing the HILM scores, understanding of key insurance concepts, and the objective knowledge dimension of the questionnaire with a subjective self-assessment question. Convergent validity was determined by comparing the scores across the different dimensions of the instrument. This analysis was inspired by the work of Tess L. C. Bardy on the translation and validation of the Swiss HILM [2].

Scores for the objective knowledge assessment were calculated by summing the number of correct responses out of 23 questions, while scores for understanding of key insurance concepts were derived from the average self-reported understanding level across 11 concepts on a scale of 1–4.

All analyses in this study were conducted using the open-source R software, with additional technical support from AI-based tools. AI tools were used primarily for code generation and syntax assistance in R, helping to streamline the implementation of statistical tests and data visualization. The selection of statistical methods, interpretation of results, and validation processes were conducted independently based on established methodologies and prior research in the field.

## Results

This section reports findings from the statistical validation stage conducted with a representative sample of respondents, as described in the methods section.

### Sample description

The questionnaire was administered online during September 2024, to 1,012 participants, with 51% identifying as women and 49% as men. The average age of participants was 46 years, with a standard deviation of 16.15. The majority of participants were Israeli-born Jews (72%), while immigrant Jews constituted 13%, and the Arab sector (including Muslim Arabs, Christian Arabs, Bedouins, and Druze) represented 15%. A significant portion of participants (69%) reported being married or living with a partner, while 21% identified as single. Regarding education, 53% held an academic degree. The presence of chronic disease was reported by 23% of participants, and 36% of participants reported taking medications regularly. 78% of participants reported using Hebrew as the primary language used at home, followed by Arabic (14%), Russian (7%), English (1%), and other languages (0.5%). In terms of employment, 70% were employed, 12% were retired, and 8% were unemployed. Detailed description of the sample characteristics is presented in Table 2.

**Table 2:** Survey participants characteristics

	*N*	%
All Sample	1,012	100
Gender		
Woman	517	51
Man	495	49
Age		
21–24	84	8
25–34	232	23
35–44	217	21
45–54	170	17
55–64	150	15
65–74	111	11
75+	48	5
Sector		
Israeli born Jews	731	72
Immigrant Jews	131	13
The Arab sector (Muslim Arabs, Christian Arabs, Bedouins, and Druze)	150	15
Family status		
Married/Living with a Partner	700	69
Single	212	21
Divorced/Widower	85	8
Single parent	7	1
Education		
Academic	540	53
Post-secondary	238	24
Secondary	216	21
Primary	18	2
Income		
Over 20 K NIS	119	12
16–20 K NIS	157	16
11–15 K NIS	304	30
5–10 K NIS	304	30
Up to 5 K NIS	128	13
Primary Occupation		
Employee	704	70
Retired	125	12
Unemployed	76	8
Self-employed	71	7
Student	36	4
Health Insurance Type		
Public, Supplemental & Commercial	419	41
No Private (Only Public)	196	19
Public & Supplemental	182	18
Public & Commercial	67	7
Don’t Remember	148	14.62

### Internal consistency and factor analysis

Table 3 outlines the mean and standard deviation of the scores of the different dimensions of the questionnaire, their Cronbach’s alphas and average inter-item correlations. Cronbach’s alpha demonstrated high internal consistency across all questionnaire dimensions (α = 0.80–0.95), with particularly strong reliability in “Confidence in choosing” (α = 0.95) and “Self-assessment knowledge” (α = 0.94), indicating that these dimensions had strong internal coherence. Average inter-item correlations varied from 0.19 for the objective knowledge assessment to 0.59 for “Behavior in choosing,” suggesting an appropriate level of association among items within each dimension.

**Table 3:** The mean score, Cronbach’s alphas and average inter-item correlation

	Scale	Mean score	SD	Cronbach’s alphas	Average inter-item correlation
HILM	1–4	2.39	0.66	0.95	0.45
Subscale 1					
Confidence in choosing		2.32	0.77	0.88	0.55
Subscale 2					
Behavior in choosing		2.33	0.80	0.91	0.59
Subscale 3					
Confidence in using		2.23	0.81	0.91	0.55
Subscale 4					
Behavior in using		2.71	0.76	0.82	0.53
Domain 1					
Confidence		2.31	0.72	0.92	0.55
Domain 2					
Behavior		2.48	0.70	0.91	0.56
Understanding of key insurance concepts		2.86	0.66	0.88	0.41
Objective knowledge assessment	0–23	10.13	5.06	0.80	0.19
Self-assessment HIL	1–10	5.08	2.04	0.94	

Scores for understanding of key insurance concepts were derived from the average self-reported understanding level across 11 concepts

Scores for the objective questions were calculated by summing the number of correct responses out of 23 questions

Self-assessment knowledge -"On a scale of 1 to 10, to what extent do you feel you understand health insurance and the health insurance market?”

Exploratory factor analysis confirmed a clear, multidimensional structure: the adapted HILM dimension replicated the original four-factor solution, accounting for 61% variance, showing clear item loadings band supporting a well-defined factor structure. The “concept understanding” dimension exhibited a coherent three-factor structure explaining 57% variance, and factor reliability was supported by a Tucker-Lewis Index (TLI) of 0.918 and RMSEA of 0.086. However, the objective knowledge assessment revealed less distinct factor loading patterns, indicating a need for future refinement to enhance clarity and thematic coherence.

The combined analysis of the entire questionnaire highlighted its multidimensional nature, with distinct factors emerging from each dimension. These factors collectively explained a cumulative 46% of the variance, with each subscale demonstrating strong item loadings and clear separations between factors. The fit indices, including a Tucker-Lewis Index of 0.912 and RMSEA of 0.037, further validate the instrument’s effectiveness in measuring the diverse dimensions of HIL. Table 4 presents the EFA results.

**Table 4:** Factor analysis results

Factor (% of variance explaining)	Question	Standardized loadings range
Factor 1 (10%)	HILM questions 1–6 Self-assessment of HIL question	43–68%
Factor 2 (7%)	HILM questions 7–12	54–73%
Factor 3 (5%)	HILM questions 13–21	49–71%
Factor 4 (6%)	Concepts questions: 2,3,5,8 & 11	50–71%
Factor 5 (6%)	Concepts questions: 6,7,9 & 10	57–70%
Factor 6 (7%)	Concepts questions: 1&4 Objective knowledge assessment questions: 1,3,4,6,9,10,16,18,20,22 & 23	31–61%
Factor 7 (5%)	Objective knowledge assessment questions: 2,5,11,12,14,15,17,19 & 21	33–57%
No factor	Objective knowledge assessment questions: 7, 8 & 13	-

### Group validity analysis

**Fig. 1:**
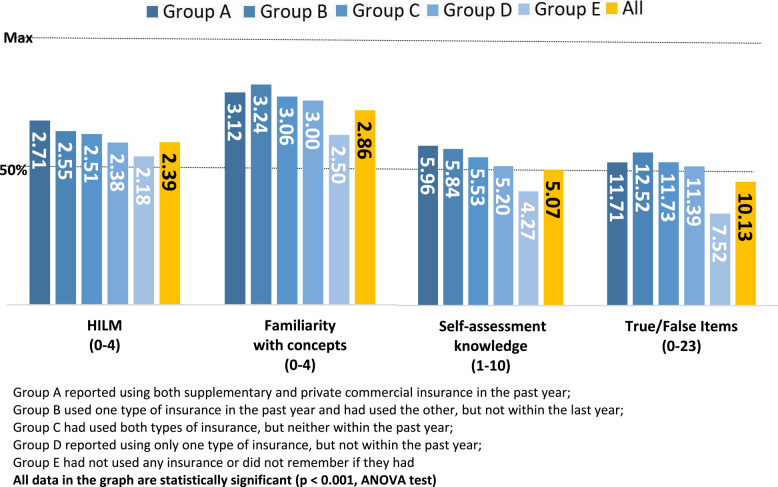
Group validity by self-reported use of health insurance

Group validity tests showed significant variation in HIL scores based on patterns of insurance usage, with active users demonstrating higher literacy scores. Group A, consisting of participants who used both supplementary and private commercial insurance in the past year, had the highest mean HILM score at 2.71 (out of 4). In contrast, the lowest mean score of 2.18 was observed in Group E, which included participants who did not use or did not remember using any insurance. Groups B, C, and D showed intermediate mean scores of 2.55, 2.38, and 2.50, respectively. The specific characteristics of Groups B, C, and D, as defined by their patterns of health insurance usage, are detailed in Fig. 1. Differences were also observed in understanding of key insurance concepts, objective knowledge assessment, and self-assessment knowledge. Figure 1 shows group validity by self-reported use of health insurance.

### Concurrent and convergent validity

Concurrent and convergent validity analyses supported the questionnaire’s ability to capture both subjective and objective HIL dimensions, reflected in moderate to strong correlations between the HILM scores and participants’ self-assessment of their health insurance knowledge (0.44 to 0.65), as well as understanding of key insurance concepts (0.47 to 0.53). Although the correlations with the objective knowledge assessment were lower, they still indicated a positive relationship (0.22 to 0.32). These results confirm that the HILM instrument effectively captures both subjective and objective aspects of HIL. Table 5 presents coefficient correlations between all dimensions of the questionnaire.

**Table 5:**
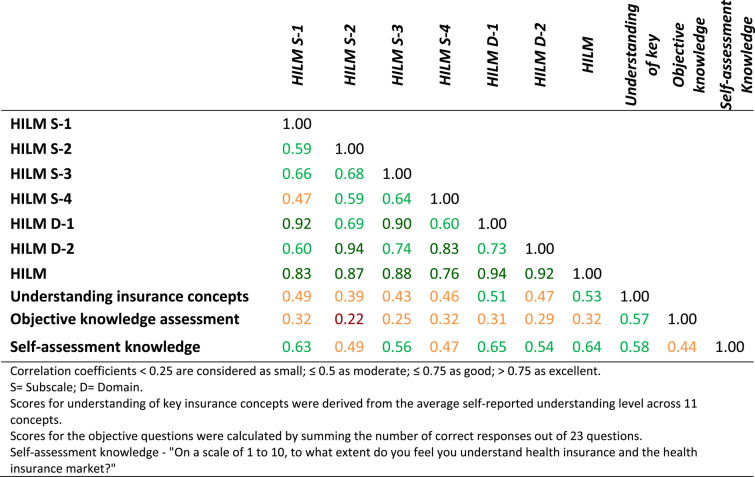
Concurrent and convergent validity - coefficient correlations

Color coding indicates correlation strength: orange for moderate (0.25 ≤ r ≤ 0.50), green for good (0.50 < r ≤ 0.75), and dark green for excellent (r > 0.75) associations, to aid interpretation

## Discussion

This study aimed to develop and validate a comprehensive measurement tool for health HIL, expanding on the HILM framework by incorporating additional dimensions and adapting it to local context, with Israel serving as a case study and proof of concept. Our findings suggest that the various dimensions of the new questionnaire effectively measure related aspects of HIL. The EFA analysis confirms the robust multidimensional structure of the questionnaire, with each dimension —HILM, objective knowledge assessment, Concepts, and Self-Reported Literacy—measuring distinct yet complementary aspects of HIL. However, the challenges in deriving a clear structure for the objective knowledge assessment highlight areas for further refinement, ensuring improved representation of objective knowledge in future iterations.

Our results of the HILM questionnaire are consistent with Bardy [2], who also reported high reliability scores for their subscales, reinforcing the notion that confidence and knowledge are interconnected components of HIL. In examining the inter-item correlations, our results ranged from 0.19 for “objective knowledge assessment” to 0.59 for “Behavior in choosing,” reflecting an appropriate level of association among items within each dimension. Similarly, Holst et al. [11] observed variable inter-item correlations in their adaptation of the HILM for the Dutch population, underscoring the need for tailored approaches to enhance clarity and relevance in assessing HIL.

Our findings on concurrent and convergent validity revealed significant associations between HILM scores and external measures included in the questionnaire. Moderate to strong correlations were observed between the HILM scores and participants’ self-assessment of their health insurance knowledge and understanding of key insurance concepts. These results confirm that the HILM instrument effectively captures both subjective and objective dimensions of HIL. Bardy [2] similarly highlighted the importance of measuring both confidence and factual knowledge, which aligns with our emphasis on understanding participants’ self-assessment in relation to their actual performance.

The lack of a clear factor structure in the objective knowledge assessment might be attributed to substantial heterogeneity among items regarding both difficulty levels and thematic coherence. Indeed, item-level performance varied considerably, with correct response rates ranging widely from as low as 17% (question_15) to as high as 77% (quation_6). This variability suggests that some items may have been overly simplistic or too complex, undermining the emergence of a consistent underlying factor structure. Additionally, certain items did not load clearly on any factor, indicating thematic inconsistency or ambiguity. Future refinement of this dimension could involve a more systematic approach to balancing item difficulty and ensuring conceptual coherence. Such modifications would enhance the reliability and interpretability of the objective knowledge assessment dimension and its relationship to the broader HIL construct.

While the correlations with the objective knowledge assessment were lower, they still indicated a positive relationship, suggesting that even these more challenging items can provide valuable insights into participants’ HIL. Holst et al. [11] also reported varied correlations across different measures, emphasizing the complexity of HIL and the influence of contextual factors on understanding.

Furthermore, the descriptive statistics from our findings provided a nuanced view of the Israeli public’s understanding and knowledge levels. Holst et al. [9] similarly reported varying levels of understanding within the Netherlands population, emphasizing that such metrics can capture the breadth of HIL across different demographics. The consistency of our findings with those of Bardy [2] and Holst et al. [11] reinforces the importance of developing HIL measures that account for both confidence and factual knowledge, while also acknowledging the contextual factors that influence the performance of different populations, including the Israeli public.

This study contributes significantly to the understanding of HIL by developing a robust and culturally adapted measurement tool. One of its primary strengths lies in the comprehensive nature of the questionnaire, which encompasses various dimensions of health insurance knowledge, including self-report confidence in understanding of key insurance concepts, factual knowledge assessed through objective knowledge questions, self-assessment, and demographic factors.

A key contribution of this study is the integration of both self-reported and objective measures of HIL. While self-efficacy is an important predictor of behavior, research indicates that self-reported confidence in understanding health insurance often does not align with actual knowledge and decision-making abilities [15]. The inclusion of factual knowledge assessments, such as objective knowledge questions and understanding of key insurance concepts, help bridge this gap and provides a more precise evaluation of individuals’ ability to navigate health insurance systems effectively. This approach aligns with recent studies that emphasize the importance of distinguishing between perceived and actual literacy to develop targeted educational interventions and policy measures [1, 4, 17].

By expanding the scope beyond the traditional HILM framework, this study offers not only a more robust and actionable tool for assessing HIL across diverse populations, but also a more efficient and informative one for policymakers. The multidimensional structure of the tool allows for pinpointing specific barriers to effective use of health insurance, thereby enabling the development of more tailored and impactful solutions.

The rigorous translation and adaptation process of the HILM ensured that the instrument accurately reflects the specificities of the contextual healthcare system, enhancing its relevance and applicability. By addressing these various aspects, this study provides valuable insights that can support advanced research in the field of HIL globally, as well as inform healthcare policy and education initiatives aimed at improving HIL.

The success of this adaptation in Israel, with its unique combination of universal coverage and supplementary insurance options, suggests that the tool can be effectively modified for healthcare systems with varying degrees of public and private sector involvement. When adapting this tool to different healthcare contexts, several key considerations should be taken. First, the basic structure of the questionnaire—combining adapted HILM items with knowledge assessment and system-specific components—can be maintained while adjusting content to reflect local healthcare delivery models. Second, the translation and cultural adaptation process demonstrated in this study, which employed both traditional back-translation and modern AI-assisted methods, provides a robust approach that can be replicated for other languages and cultural contexts. Third, the tool’s modular design allows for selective modification of dimensions while maintaining core elements that enable cross-national comparisons. For example, while the HILM component provides a standardized base for international comparison, the knowledge assessment dimension can be customized to reflect country-specific regulations, coverage structures, and healthcare delivery systems. Additionally, the validation process described here—incorporating expert review, pilot testing, and psychometric analysis—offers a systematic approach that can be replicated across different healthcare contexts.

Despite the comprehensive nature of this study, several limitations should be acknowledged. One significant limitation is the reliance on self-reported measures in dimensions of the questionnaire, which may introduce biases, as participants might overestimate their understanding of health insurance concepts. While the inclusion of objective knowledge assessment questions helps mitigate this issue by objectively assessing knowledge, the extent of participants’ understanding still relies on their self-assessment in other areas. Additionally, although the sample represents diverse demographics within the Israeli public, it may not fully capture the nuances of all socio-economic groups or regions, potentially limiting the generalizability of the findings. Lastly, the cross-sectional design of the study constrains our ability to assess changes in HIL over time, which could be crucial in understanding the impact of evolving healthcare policies and practices.

This study makes a significant contribution to the field of HIL by presenting a comprehensively validated measurement tool that bridges global and local dimensions of HIL. Building upon existing literature, our research extends the understanding of HIL by emphasizing the critical importance of capturing both universal core skills and local contextual nuances. By integrating global competencies and system-specific factors, we contribute to a more comprehensive approach that recognizes HIL as a multidimensional construct. Our methodology acknowledges that HIL is not just about universal skills like self-efficacy and insurance navigation, but also about understanding how socioeconomic status, cultural background, and local regulatory environments profoundly mediate health insurance comprehension and utilization, as also suggested by Holst et al. [10].

In line with previous research in the field, we provide a robust, adaptable framework that enhances our understanding of HIL. By developing a tool that captures the nuanced interactions between objective knowledge, subjective confidence, and systemic understanding, we offer a model that can inform future research and policy initiatives aimed at improving health insurance comprehension across different populations and healthcare systems.

Additionally, the multidimensional nature of our questionnaire, which evaluates knowledge, comprehension, confidence, and practical skills —highlights the necessity of broadening current approaches to HIL interventions. While traditional educational interventions predominantly focus on enhancing factual knowledge, our findings suggest that addressing dimensions such as confidence, comprehension, and practical navigation skills may be equally, if not more, critical. Consequently, policymakers and educators should consider interventions such as practical workshops, scenario-based simulations, or personalized consultations aimed at building individuals’ confidence and practical competencies in using health insurance effectively. Such multidimensional strategies could yield more substantial improvements in health insurance utilization than knowledge-centric interventions alone.

## Conclusions

This study successfully developed and validated a comprehensive measurement tool for health insurance literacy (HIL), designed to address global HIL dimensions while being adaptable to diverse cultural and national contexts, with Israel serving as a case study and proof of concept. By expanding upon the HILM framework, the research contributes to a more nuanced understanding of the factors shaping individuals’ ability to navigate complex healthcare systems.

Our findings highlight the importance of incorporating both universal competencies and context-specific nuances when measuring HIL. The robust internal consistency, valid correlations, and multidimensional structure of the tool align with prior studies such as Bardy [2] and Holst et al. [11], emphasizing the value of contextually adapted instruments that maintain comparability across diverse populations.

The comprehensive nature of the questionnaire, integrating self-reported measures with objective knowledge assessments, underscores its applicability in both academic and practical settings. This tool offers a foundation for future research aimed at understanding and addressing HIL globally while providing actionable insights for policy development and educational interventions. By promoting improved HIL, this work supports efforts to enhance equitable access to healthcare and improve health outcomes across populations.

## Supplementary Information

Supplementary Material 1.

## Data Availability

The data supporting the findings of this study are available from the corresponding author upon reasonable request. The full dataset is held by the author and can be provided upon inquiry.

## Abbreviations

HIL: Health Insurance Literacy
HILM: Health Insurance Literacy Measure
