# Supplementary material for: Development and validation of a new comprehensive measurement tool for health insurance literacy
Source: BMC Health Serv Res. 2025 Dec 8;25:1580. doi: 10.1186/s12913-025-12881-9 (PMC12687537; doi:10.1186/s12913-025-12881-9)
Supplement: Supplementary file 1 — Supplementary Material 1. [file 12913_2025_12881_MOESM1_ESM.pdf]

Israeli Health Insurance Literacy Questionnaire

Chapter One - Skills and Capabilities in Using Health Insurance

(Translation and Adaptation of HILM Questionnaire)

How confident or not confident are you ...

	Not at all sure	Somewhat sure	Fairly sure	Very sure
1. You understand the concepts related to health insurance in Israel				
2. You understand the differences between HMO supplementary insurance and private commercial health insurance				
3. You can estimate your monthly insurance cost for next year (excluding emergencies)				
4. You know what questions to ask to choose the right health insurance for you and your family				
5. You know where to find the information needed to choose the right health insurance for you and your family				
6. You will succeed in choosing the most suitable health insurance for you or your family at the HMO or with a commercial insurance company				

When you want to purchase a new health insurance plan and you're comparing different health insurance plans, how likely are you to understand...

	Not at all likely	Somewhat likely	Quite likely	Very likely
7. If the insurance policy covers unexpected costs, such as rehabilitation after an accident or stroke				
8. How much you'll need to pay for private hospital surgery				
9. How much you'll need to pay out of pocket for visiting a specialist not through the HMO				
10. How much you'll need to pay for prescription medications not in the national health basket				
11. Which doctors and hospitals are covered by the insurance policy				
12. What are the differences between various insurance policies offered to you				

When using or activating your health insurance at HMOs/private insurance company, and before receiving certain treatment, how confident do you feel that...

	Not at all sure	Somewhat sure	Fairly sure	Very sure
13. You'll know how to check what is and isn't covered by your health insurance before receiving treatment				
14. You'll know what to do if the insurer refuses to pay or reimburse for treatment you think should be covered				
15. You'll succeed in understanding the cost-sharing between out-of-pocket payment and insurance coverage				
16. You'll know what questions to ask if you have a problem with coverage or reimbursement				
17. You'll know most details required to use your health insurance				

Please indicate how likely are you to act in the following ways when using or activating your health insurance at HMOs/private insurance company before receiving certain treatment...

	Not at all likely	Somewhat likely	Quite likely	Very likely
18. Contact customer service of the HMO and/or private insurance company to ask which treatment is covered by your health insurance				
19. Succeed in understanding what is and isn't covered by your insurance policy before certain treatment				
20. Check if certain treatment or doctor is included in your insurance policy before starting treatment				
21. Read and check the quarterly bill you receive from the HMO or private insurance company				

Chapter Two - Familiarity with Concepts in the Israeli Health Insurance Market

For the following concepts in health insurance, please indicate what applies to you:

	Never heard of this concept	Heard of the concept but don't understand it	Know the concept and understand it in context, but can't explain it	Understand it well and can explain its meaning
22. National Health Insurance Law				
23. Supplementary Health Insurance				
24. Health Declaration				
25. Qualification Period				
26. Deductible/Copayment				
27. Actuary				
28. Underwriting				
29. Pre-existing Condition				
30. Premium				
31. Changing Premium				
32. Health Basket				

Chapter Three - True/False Knowledge Questions

Please note:

- "Supplementary Health Insurance" refers to additional health services in HMOs (like Clalit Mushlam and Platinum, Maccabi Gold and Silver and Maccabi Sheli, etc.)
- "Private-Commercial Health Insurance" refers to commercial insurance (private or group) from insurance companies like Harel, AIG, Migdal, Menora, etc.

For each statement, please indicate if it is True or False regarding the current Israeli healthcare system:

	True	False	Don't Know
33. Public-national health insurance is funded by health insurance tax (collected by National Insurance Institute) and supplemented by state budget sources			
34. In addition to health tax, payment for national (public) health insurance is paid monthly directly by the insured to the HMO			
35. Payment for supplementary health insurance is made according to usage only			
36. Payment for supplementary health insurance is made according to a fixed monthly premium plus copayment for services used			

37. The HMO may refuse to provide health services included in the service basket to a member who hasn't paid or is late for insurance payments			
38. Every Israeli resident is legally entitled to receive any treatment or medication included in the national-public health basket (based on medical need)			
39. A person who doesn't pay monthly payments to the HMO can visit a family doctor for free or for a low quarterly or one-time copayment			
40. A person who doesn't pay directly to the HMO monthly can seek additional consultation (second opinion) with a specialist for free or for a low quarterly or one-time copayment			
41. Arriving at the ER without professional referral is free or costs a low quarterly or one-time copayment			
42. Surgery in a private hospital allowing a choice of surgeon is included in public insurance rights			
43. Child development treatments are only given to those with supplementary or private insurance			
44. Fertility treatments are only given to those with supplementary or private insurance			
45. Alternative treatments (complementary medicine) are only given to those with supplementary or private insurance			
46. To purchase national-public insurance, you must sign a health declaration			
47. To purchase supplementary insurance, you must sign a health declaration			
48. To purchase private-commercial insurance, you must sign a health declaration			
49. In supplementary insurance, the HMO can decide to terminate the insurance agreement on its own initiative			
50. In private-commercial health insurance, the insurance company can decide to terminate the insurance agreement on its own initiative			
51. In supplementary insurance, the HMO can decide not to insure a person suffering from a chronic illness or above a certain age			
52. In private-commercial health insurance, the insurance company can decide not to insure a person suffering from a chronic illness or above a certain age			
53. In national-public health insurance, using some insurance coverages requires waiting for a qualification/waiting period from policy purchase			
54. In supplementary insurance, using some insurance coverages requires waiting for a qualification/waiting period from policy purchase			
55. In private-commercial health insurance, using some insurance coverages requires waiting for a qualification/waiting period from policy purchase			

Chapter Four - Self-Assessment

56. To what extent do you feel you understand or don't understand health insurance and the health insurance market in Israel? On a scale of 1 - "Don't understand at all" to 10 - "Understand excellently"

Chapter Five - Demographic Questions + Insurance Use

57. Gender
58. Birth year
59. Sector/Religion
60. Level of religiosity
61. Marital status
62. Children: Yes or No
63. Living area
64. Education
65. Income
66. Country if birth and immigration year
67. Employment status
68. What is the main language you speak at home today?
69. Please rate your health condition relative to your age between 1-10 where 1 means "not good at all" and 10 means "very good"
70. Do you have any chronic illnesses? - Yes / No
71. Do you take medications regularly? - Yes / No
72. Which HMO are you insured with?
73. What health insurance do you have?
74. Have you ever used the benefits in supplementary insurance?
75. Have you ever used/requested reimbursement from your private-commercial health insurance?
